# Acetaminophen, a new tool for the refinement of the experimental infection of toxoplasmosis in mice

**DOI:** 10.1038/s41598-025-06849-2

**Published:** 2025-07-01

**Authors:** Nathalie Moiré, Axel Cauty, Christelle Rossignol, Corinne Beaugé, Laetitia Mérat, Emilie Lortscher, Françoise Debierre-Grockiego, Isabelle Dimier-Poisson, Mickaël Riou

**Affiliations:** 1 INRAE-Université de Tours, UMR-1282 Infectiologie et Santé Publique (ISP), Équipe BIOMAP, UFR des Sciences Pharmaceutiques, 31 Avenue Monge, 37200 Tours, France; 2 INRAE-Université de Tours, UMR-1282 Infectiologie et Santé Publique (ISP), Équipe IMI, Centre Val de Loire, 37380 Nouzilly, France; 3 INRAE, UE-1277 Plateforme d’Infectiologie expérimentale (PFIE), Centre Val de Loire, 37380 Nouzilly, France

**Keywords:** Welfare, Acetaminophen, Toxoplasmosis, Mice, Behavior, Immune response, Adaptive immunity, Innate immunity, Acute inflammation, Chronic inflammation, Medical ethics

## Abstract

In recent years, animal welfare gained increasing importance in society and especially in scientific research. It has become necessary to refine the experimental procedures as much as possible in infectiology as in our reference model, toxoplasmosis, in accordance with the 3Rs rule and various ethical concerns. Thus, the establishment of a treatment using analgesics would provide relief to animals acutely infected with *Toxoplasma gondii*. However, the use of analgesics should in no way alter the pathophysiology of the disease and the host immune response, so as not to interfere with the initial scientific study. Little is currently known about the use of acetaminophen (APAP) in an infectious model. In the present work, we studied the impact of APAP at a reference dose of 30 mg/kg/day in a mouse model of acute toxoplasmosis. Zoonotic, telemetric, behavioral, histological and immune parameters were analyzed to better characterize the consequences of treatment with APAP either by gavage or by self-medication in Gel Water. APAP administered by gavage did not induce cellular or tissue toxicity or alter the physiological development of the mice. In addition, the nature of Gel Water itself, independent of APAP, had an effect on the immune response. APAP improved overall well-being and slowed the onset of clinical signs without altering the physiopathology or the immune responses induced by *T. gondii*. These first results in mice confirmed our initial hypothesis that APAP appears to be a pharmacological tool to refine and improve animal welfare during the acute phase of toxoplasmosis. Therefore, our project has highlighted the combination of specific markers to contribute to animal welfare in mice. In the long term, the use of APAP could be extended to other infectious models with other target animal species.

## Introduction

In recent years, animal welfare has become increasingly important in society, especially in scientific research. Since 2013, a European decree has been established regarding the use of animals in experimental procedures, and the need for precise ethical evaluation of each project to be carried out. This involves strengthening ethics committees but also reminding and highlighting a rule that has been in place since 1959: the 3Rs^1,2^. The objectives of this rule are multiple and therefore go through three main points: To Reduce as much as possible the number of animals used during experiments and to Replace animal models with in vitro or bioinformatics models whenever possible. A key point of this rule, to Refine, refers to methods that minimize the pain, suffering, distress that may be experienced by research animals, and which improve their welfare. Pain and distress can alter the behavior, physiology and immunology of animals, leading to variations in experimental results that may affect the reliability of studies^3^.

Refinement can include an enrichment of animals’ environment, such as game or reward which can improve overall well-being^4,5^ and the introduction of endpoints that allow humans to assess animal suffering. Finally, pain management using analgesics and/or anti-pyretics also has an impact on scientific results due to the choice on appropriate analgesics or lack of pain management. The use of drugs is not so straightforward in animal experiments, especially in infectious disease models because, of the influence that pharmacological substances may have on the results of the study. A dilemma then arises because symptoms related to an infection that can lead to the death of the animal could be limited with the appropriate medication. However, untreated pain can also affect, by example, the immune system. Several reports demonstrate that stress associated with pain may lead to immunosuppression^6^. In all cases experimental bias may occur^7^. Before being able to generalize the use of drugs to relieve animals, it is necessary to know their mechanisms of action, the possible toxicity they could induce, but also their influence on the immune response and whether a cause-and-effect relationship to the infectious agent used can be observed.

Opioids and nonsteroidal anti-inflammatory drugs (NSAIDs) are the two main groups of pain relievers available^8^. Buprenorphine and meloxicam are the most commonly used analgesics in rodents and extensive literature review of pharmacokinetics of these drugs are available. Although it is one of the most largely used analgesic/antipyretic drug in the world for human use^9^, acetaminophen (N-acetyl-p-aminophenol or APAP) is rarely used to treat rodents. It is known to have no anti-inflammatory effect, unlike other nonsteroidal anti-inflammatory drugs^10^. The mechanism by which it produces its analgesic effect is largely unknown^11^. It is supposed that APAP would inhibit the synthesis of prostaglandins comprising a COX site (active site of the majority of NSAIDs^12^) in the central nervous system, prostaglandins having a pro-nociceptive role and potentially causing fever in the hypothalamus on which APAP would act^13^. Similar to its analgesic action, the mechanism of antipyretic action of this drug remains poorly understood.

However, its administration is easily done orally and its absorption by oral route is complete and rapid, as the maximum plasma concentration is reached within one hour of ingestion^14^. Following oral administration, its systemic bioavailability is dose-dependent and ranges from 70 to 90% with differences between animal species^15^. APAP is distributed rapidly in all tissues and is quickly eliminated by reaction with reduced glutathione and then excreted in the urine after conjugation with cysteine and mercapturic acid^16^. Nevertheless, literature studying APAP use in experimental infectious rodent models is rare and most use concern pain relief after severe invasive procedures (in combination with opioids) or in cancer model.

Here, we were more interested in the antipyretic and analgesic effect of APAP and how this could reduce overall stress and unease during the acute phase of an infectious disease. For this study, we focused on a murine toxoplasmosis model. *Toxoplasma gondii*, the causative agent of toxoplasmosis, is an obligate intracellular protist that is ubiquitous and exhibits a wide host range^17^. The parasite undergoes three distinct stages of development within its host: tachyzoites, which rapidly proliferate during the acute phase of infection; bradyzoites, which persist within latent cysts in tissue; and sporozoites, which reside within oocysts, a form of resistance in the environment^18^. Initial contact with the parasite triggers a protective immune response in immunocompetent animals or humans with no, or only few, symptoms. However, the infection can lead to clinical symptoms (weight loss, temperature drop, prostration…) and death in some mouse models, depending on the infectious dose. *T. gondii* induces a pro-inflammatory (TH1 type) immune response, mediated by IFN-γ and IL-12 production^19,20^ by T lymphocytes and antigen-presenting cells, respectively. This immune response is known to control the parasite in the acute phase, which allows it to persist in different tissues and organs, primarily in the brain, as dormant cysts, resulting in chronic infection. The transient inflammation that is characteristic of the acute phase of toxoplasmosis has been identified as a potential cause of clinical symptoms.

The aim of the study was to determine whether treatment of infected mice with APAP could relieve the pain or discomfort induced during the acute phase of toxoplasmosis without altering parasite dissemination and immune response, while improving the welfare of the animals. To date, only one publication has described the use of buprenorphine to relieve pain and distress following acute *T. gondii* infection^21^. In the present work, the safety of APAP was tested in CBA/J mice at the dose of 30 mg/kg/day, by gavage or by self-administration. This dose is recommended by the supplier of this veterinary drug already used in pigs^22,23^.

The same treatment was applied to CBA/J mice infected with *T. gondii* in order to study the effects of APAP on the pathophysiology (parasite multiplication and establishment) of the infection model and the specific humoral and cellular immune responses. Zootechnical (temperature, weight, behavior), immunological, biochemical and histological analyses were also performed. The main objective of the whole study was to validate the proof of concept for the use of APAP as a pharmacological tool to refine animal experiments using the murine model of toxoplasmosis.

## Materials and methods

### Animals and ethics statement

Experiments were carried out according to EU directives and French regulations (Directive 2010/63 / EU, 2010; Rural Code, 2018; Decree n ° 2013–118, 2013, https://www.legifrance.gouv.fr/loda/id/JORFTEXT000027038013/) and with ARRIVE guidelines. All experimental procedures have been evaluated and approved by the Ministry of Higher Education and Research (APAFIS # 2018021917268751.V3—13634). The procedures involving mice were evaluated by the Val de Loire ethics committee (CEEA VdL, committee number 19) and took place at the INRAE Platform for Experimental Infection PFIE (UE-1277 PFIE, INRAE Centre-Val-de Loire) Valley research, Nouzilly, France, https://doi.org/10.15454/1.5535888072272498e12).

A total of 90 female mice of the CBA/J line (Janvier Labs, Le Genest-Saint-Isle, France) aged 5 weeks at the start of the experiments were used. Mice were randomly identified using telemetric sensors (Biolog-Animal®, Paris) implanted subcutaneously in the dorsal region, under general anesthesia with 4% isoflurane (Vetflurane®, Virbac, France). At the implantation area, a small amount of Tronothane® 1% in Gel form (DELPHARM, L’Aigle) was applied to relieve the animal. After injection, a small massage at the injection site was performed to maintain the chip. These telemetric sensors allow individual monitoring and temperature measurement added to general condition, weight, and behavior throughout the protocols. Mice were housed in groups of 4 to allow social interaction in T2 type cages on bedding and in an enriched environment on a Techniplast ventilated rack (Techniplast, Louviers). Humidity (between 45 and 65%) and temperature (between 20°C and 24°C) were controlled daily. The light cycle is 12/12. Mice were followed by daily zootechnical visits with a checklist of previously clinical signs allowing quotation of welfare (Table 1). All clinical observations were made in the morning, starting with temperature reading using a chip reader, followed by weight reading and finishing by behavioral tests. Mice were always observed in the same order.

**Table 1:** Description of clinical signs and quotation following infection with *T. gondii* during the acute phase for the assessment of well-being.

Quotation	Clinical signs
0	No symptom
1	Increase or drop in temperature or weight loss compared to D0 (< 36.5° or > 38.2° C)
2	Weight loss and/or temperature drop compared to D0 and/or tousled hair
3	Weight loss + temperature drop + tousled hairs or almond-shaped eyes (beginning of facial tension) or low vibrissae or prostration
4	Weight loss + temperature drop + tousled hairs and/or low vibrissae and/or almond-shaped eyes (facial tension and/or ears back) and/or prostration
5	Weight loss + temperature drop + tousled hairs + low vibrissae + almond eyes (face tightness, ear back) + prostration

Quotation 5 corresponds to the limit points reached, leading to the animal’s death.

### Experimental designs

**Fig. 1:**
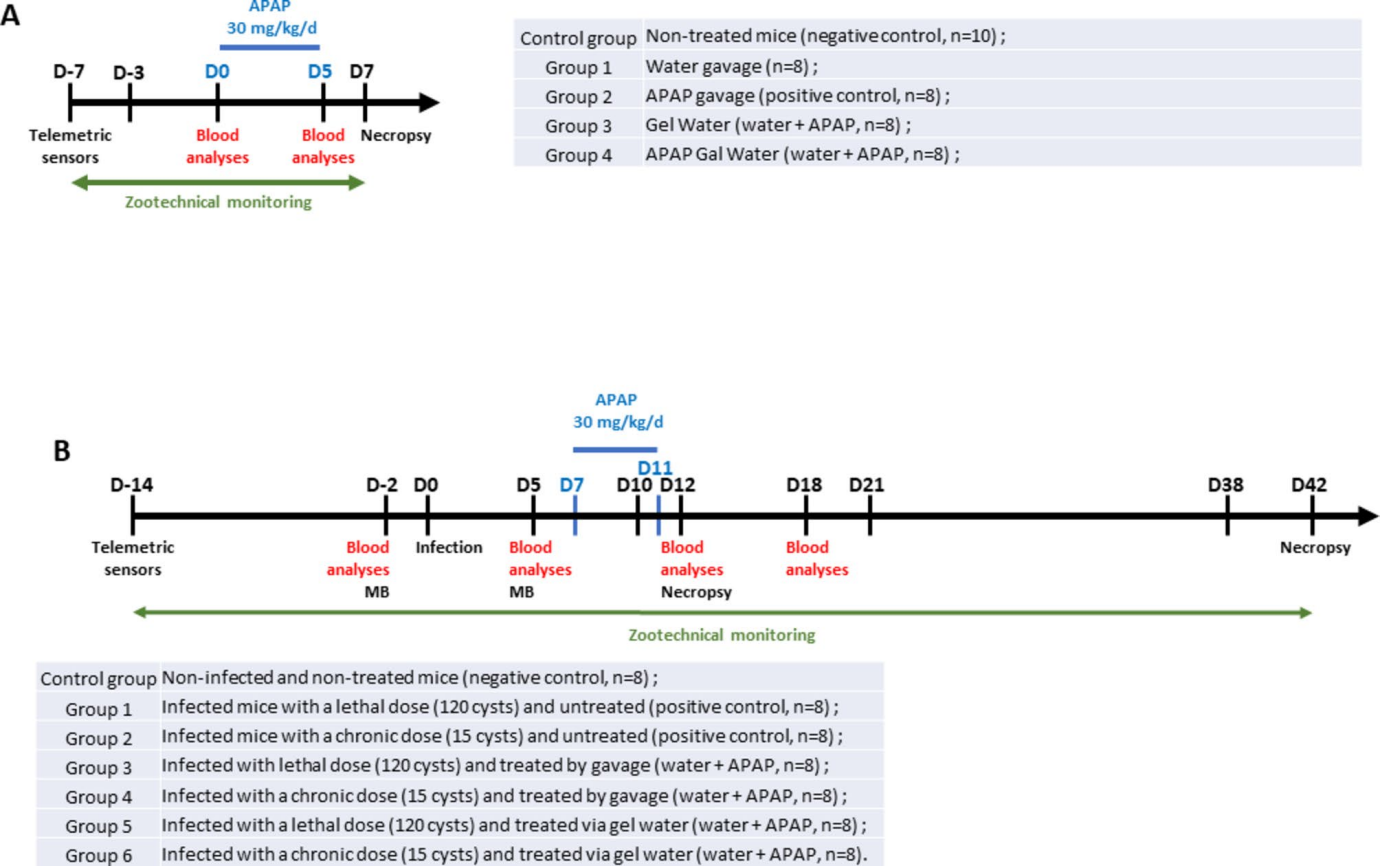
Experimental timelines. (**A**) Evaluation of APAP cytotoxicity in naïve CBA/J mice. (**B**) Effect of APAP on *Toxoplasma gondii* pathophysiology (acute and chronic phases) and impact on the immune response and behavior in CBA/J mice. *MB* Marble burying test.

Acetaminophen (N-acetyl-p-aminophenol or APAP) was administered to non-infected mice (first experiment) and to mice infected with *T. gondii* cysts (second experiment) for five consecutive days (Fig. 1A and B). Blood samples were taken and zootechnical data collected throughout the experiments. Organs (spleen, liver, kidneys, stomach, brain) were sampled at the end of the experiments for histological analyzes.

### Oral administration of APAP

APAP solutions were prepared every morning for 5 days of treatment before their administration. For gavage, 75 µL of 40 % Pracetam® (400 mg/mL, CEVA Santé Animal, Libourne, France) were mixed in 9925 µL of water to obtain the correct dose (30 mg/kg/day according to the recommendations of the SAFE complete care competence company). For self-ingestion, 4 doses of Safe® Geldiet Water (Safe SAS) corresponding to 400 mL were liquefied by heating in a microwave for 2 min and mixed in a beaker by magnetic stirring. Then, 12 μL of Pracetam® were added and after homogenization, the content of the beaker was transferred into the original jars, identified, weighed and solidified at 4°C for one hour. Treatment (30 mg/kg/day) was held over a period of 5 consecutive days. For gavage, mice were force-fed with 200 µL of water or water + APAP. For self-medication, one dose of Gel Water or Gel Water + APAP was placed on the floor of each cage of 4 mice and weighted at 24 h to monitor consumption. In Gel Water, APAP concentration is identical. However, the dose ingested by the mice is different, because in Gel Water, the mice self-administer the drug to mimic natural mouse behavior. In all conditions, water was available *ad libitum.*

### Mice behavior using Marble burying test (anxiety test)

The Marble Burying test/anxiety test^24^ was performed regularly (D-2 = 2 days before infection, D5, D10, D21, D38 pi). Each mouse was placed individually in a T3 type cage filled with 3 cm of litter for 5 min in order to get used to the new environment. The mouse was removed, then 15 beads (1.6 cm diameter) were placed in 5 rows and 3 columns. The mouse was returned to the cage and a score was assessed on each bead until 20 min (intact marble = 0, partially buried = 1, buried but visible = 2, completely buried = 3).

### Infection of mice by gavage with T. gondii cysts

The cysts were obtained from brains of CBA/J mice infected by gavage 2 months before with the *T. gondii* strain 76K. Mouse brains were homogenized in 5 mL of RPMI 1640 medium, and the number of tissue cysts per brain was determined microscopically by counting 3 samples (10 µL each) of each homogenate. The “lethal dose” groups received 120 cysts while the “chronic dose” groups received 15 cysts in the final volume of 200 µL by gavage^25^. The mice receiving 120 cysts were euthanized by cervical dislocation without anesthetic before day 12 of infection prior to reaching the end point previously defined and the end of protocol for all other mice.

### Blood tests for serum monitoring and biochemical markers analysis

Blood samples (120 µL final serum) were taken from the mandibular vein with a sterile lancet on a sterile 2 mL dry tube. Blood samples were pooled as mouse pairs which stayed the same throughout the study. Cytotoxicity of APAP was analyzed by analysis of biochemical parameters of target organs: liver (alanine aminotransferase, alkaline phosphatase, aspartate aminotransferase), kidneys (creatinine and urea) and glucose as a general parameter. Serum samples (diluted 1/10 and 1/61 in M-Scan II diluent) were placed in Select-6V crowns and analyzed using the M-ScanII Biochemical analyzer (Melet Schloesing Laboratories, France) as previously described^26^.

### Organ histology

Spleen, brain, lung and stomach tissues fixed in 4 % formalin (Carbo-Erba Reagents, Val de Breuil) for two weeks were embedded in paraffin wax (Paraplast plus, Leica) using an automatic device (TP1020, Leica). With a manual rotary microtome (RM2235, Leica), tissue sections 5 μm thick were deposited on Superfrost plus® slides (Thermo Fisher Scientific, Artenay) before being dried at 37° C overnight. The usual topographic staining by Haemalun-Eosin was used to observe the tissue structure of the different samples. The tissues were kept between slides and coverslips to be observed under a microscope (Eclipse 80i, Nikon).

### Cell staining

Single-cell splenocyte suspensions were obtained from spleen first pressed and then filtered through a nylon mesh. Hypotonic shock (0.155 M NH_4_Cl, pH 7.4) was used to remove erythrocytes. The cells were then suspended in RPMI 1640 medium supplemented with 5% fetal calf serum (FCS), 25 mM HEPES, 2 mM L-glutamine, 1 mM sodium pyruvate, 50 µM 2-β-mercaptoethanol, and 1 mM penicillin–streptomycin and counted. Splenocytes were seeded at 5.10^5^/200 µL into 96-well round-bottom culture plates. After centrifugation for 5 min at 700 g, the supernatants were discarded and 100 μL of each antibody diluted in PBS 5% FCS were added. Antibodies for the detection of CD4 (clone GK1.5), CD8 (clone eBioH35-17.2), CD19 (clone ebio1D3) and F4/80 (clone BM8) and their respective isotype were purchased from eBioscience and cells stained as described^27^.

The cells were homogenized and the plates incubated for 30 min at 4 °C. The plates were centrifuged, the supernatants removed and cells were washed with 100 μL of PBS containing 5% FCS. This step was repeated, then 100 μL of 2 % paraformaldehyde (w/v) in PBS were added and the plates were placed at 4° C until analysis of 10,000 events by flow cytometry (MACSquant, Miltenyi).

### Cytokine quantification

For cytokine detection, splenocytes were recovered and purified as described above. Splenocytes were seeded at 5.10^5^/200 µL medium into 96-well flat-bottomed plates and stimulated for 72h with 10 µg/mL *T. gondii* total soluble extract (TE) or with concanavalin A at 5 µg/mL as previously described^27^. Levels of mouse cytokines were quantified in the culture supernatants by using IL-6, IFN-γ, IL-12p40 specific sandwich enzyme-linked immunosorbent assay (ELISA) following the manufacturer’s instructions (cytokine mouse uncoated ELISA kit, Invitrogen) as described^27^.

### Detection of anti-toxoplasmic immunoglobulins-G in serum by ELISA

Titers of *T. gondii*-specific IgG antibodies were performed by ELISA on sera. Flat-bottomed 96-well plates (Nunc) were coated overnight with 10 µg/mL TE in 50 mM carbonate buffer (pH 9.6). The plates were washed with PBS-Tween 0.05% and blocked with PBS-4% bovine serum albumin (BSA, Sigma-Aldrich). Serial dilutions of serum were performed in PBS-BSA 4%, and the plates were incubated for 2h at 37 °C. The plates were then washed again and incubated 1h at 37 °C with goat anti-mouse IgG alkaline phosphatase (1:5000, Sigma-Aldrich). After washes, para-nitro-phenyl-phosphate (Sigma-Aldrich) diluted in DEA-HCl at 10 mg/mL was added. The absorbance of each sample was measured at 405 nm. Titers of IgG antibodies were determined as the highest serum dilution that exhibited an absorbance at least twice that of the mean absorbance of eight wells containing the negative control serum.

### DNA extraction and qPCR

DNA was extracted from 25 mg tissue (brain, lungs or spleen) using Nucleospin tissue extraction kit (Macherey–Nagel). Quantitative PCR (qPCR) was performed on 200 ng of genomic DNA in a total volume of 20 µl containing LightCycler® Taqman® Master mix (Roche Diagnostics), 0.5 µM of 2 primers (TG III: 5’-CCT TGG CCG ATA GGT CTA GG-3’; TG IIb: 5’-GGC ATT CCT CGT TGA AGA TT-3’, and 180 nM of the probe FAM-5’-FAM-TGC AAT AAT CTA TCC CCA TCA CGA TGC ATA CTC AC-TAMRA-3’ (Eurofins Genomics, France). The qPCR program was 2 min at 50°C, 5 min at 95°C, 50 cycles of 20s at 95°C/60s at 65°C with the LightCycler® 2.0 Instrument (Roche Diagnostics, France). The target sequence codes for ribosomal RNAs and is present as rDNA repeating units^28^.

Standard curves were generated with DNA of known amounts of tachyzoites alone or extracted with the DNA of brain, lungs or spleen of non-infected mice as described^29,30^.

### Enumeration of brain cysts

Brains were homogenized in 5 mL of RPMI 1640 medium in a glass potter, and the number of tissue cysts per brain was determined microscopically by counting 10 samples (10 µL each) of each homogenate.

### Statistical analyzes

Statistical analyzes were performed using GraphPad Prism software, version 6.0 (GraphPad, San Diego). Based on distribution, tested using Bartlett test, nonparametric tests Kruskal–Wallis (followed by a Dunn’s multiple comparisons test) or parametric tests were performed.

A one or two-way ANOVA analysis (followed by Tukey or Sidak’s post-test) were performed to show the effects of treatments on the measured parameters. Non-parametric Kruskal Wallis tests were carried out to analyze cytokine response, parasite load and cell populations. All statistical tests were two-sided and a value of P < 0.05 was considered statistically significant.

## Results

### Lack of toxicity of APAP treatment in non-infected CBA/J mice

The temperature and weight of the mice were monitored from D-3 to D7, with APAP treatment from D0 to D5 (Table 2). Before treatment, mice had a mean weight between 18.80 ± 0.92 and 20.13 ± 0.99 g. The mice gained weight in accordance with the reference growth curve of the CBA/J strain, with no significative difference between groups, treated or not with APAP. Likewise, no significant variation has been observed for body temperature, which varied between 37.27 ± 0.69 and 38.40 ± 0.68 °C.

**Table 2:** Zootechnical monitoring of CBA/J mice during the APAP treatment phase (weight/temperature, D-3, D0, D5, D7, expressed as mean ± standard deviation, corresponding to n = 8 mice /group and n = 10 mice for control group).

	Day -3		Day 0		Day 5		Day 7	
	Weight (g)	Temperature (°C)	Weight (g)	Temperature (°C)	Weight (g)	Temperature (°C)	Weight (g)	Temperature (°C)
Control	18.80 ± 0.92	37.56 ± 0.33	19.20 ± 0.92	37.81 ± 0.63	19.70 ± 1.49	38.20 ± 0.70	19.80 ± 1.48	38.40 ± 0.68
Water by gavage	20.13 ± 0.99	37.86 ± 0.41	19.50 ± 1.41	37.29 ± 0.61	20.25 ± 1.16	37.56 ± 0.0.66	20.25 ± 1.28	37.76 ± 0.71
APAP by gavage	20.00 ± 1.20	37.80 ± 0.47	20.13 ± 0.99	37.90 ± 0.29	20.50 ± 1.41	38.03 ± 0.53	21.00 ± 1.20	37.34 ± 0.76
Gel water	19.13 ± 1.13	37.31 ± 0.50	18.88 ± 1.64	37.39 ± 0.45	19.25 ± 1.16	36.63 ± 0.55	20.25 ± 1.67	37.65 ± 0.47
APAP + Gel water	19.75 ± 1.28	37.40 ± 0.35	20.50 ± 1.31	37.44 ± 0.61	20.13 ± 0.99	37.44 ± 0.67	20.88 ± 1.55	37.27 ± 0.69

For each blood sample, biochemical quantification of alkaline phosphatase, aspartate transaminase, alanine aminotransferase, urea and glucose (Table 3) was carried out to assess the potential toxicity of APAP on the organs involved in its degradation and elimination (liver, kidneys). Except for elevated levels of alkaline phosphatase and three controls at D0 for ASAT, all values were within the range of norms given by the supplier for the mouse species. For all parameters quantified, no significant difference between the groups and in comparison, to the day before the treatment was observed, except three control groups for ASAT. Creatinine was measured but not detectable (< 1.9 mg/mL).

**Table 3:** Monitoring of serum biochemical parameters, indicators of drug toxicity in CBA/J mice.

	Groups	ALP (U/L)	ASAT (GTP, U/L)	ALAT (GOT, U/L)	Urea (g/L)	Glucose (g/L)
Day 0	Control	332 ± 23	85 ± 18	23 ± 2	0.34 ± 0.05	1.22 ± 0.22
	Water by gavage	236 ± 56	69 ± 24	9 ± 3	0.41 ± 0.04	1.72 ± 0.32
	APAP by gavage	250 ± 52	94 ± 27	32 ± 11	0.40 ± 0.05	1.51 ± 0.45
	Gel water	294 ± 29	102 ± 39	22 ± 6	0.41 ± 0.03	1.53 ± 0.03
	APAP + Gel water	265 ± 50	66 ± 21	34 ± 3	0.42 ± 0.03	1.89 ± 0.19
Day 5	Control	234 ± 37	94 ± 21	41 ± 12	0.39 ± 0.05	1.60 ± 0.21
	Water by gavage	192 ± 34	132 ± 12	41 ± 3	0.40 ± 0.09	1.60 ± 0.15
	APAP by gavage	218 ± 40	88 ± 27	38 ± 6	0.37 ± 0.03	1.63 ± 0.28
	Gel water	271 ± 37	105 ± 10	43 ± 2	0.33 ± 0.04	1.39 ± 0.15
	APAP + Gel water	193 ± 6	106 ± 22	42 ± 2	0.41 ± 0.03	1.70 ± 0.11
Range of normal values (min–max)		62–209	59–247	28–132	0.38–0.67	0.9–1.92

(n = 4 serum samples per group, except 5 serum samples for the control, corresponding to n = 8 mice /group and n = 10 mice for control group multiple comparison test between D0 and D5).

*ALP* alkaline phosphatase, *ASAT* aspartate transaminase, *ALAT* alanine aminotransferase.

**Fig. 2:**
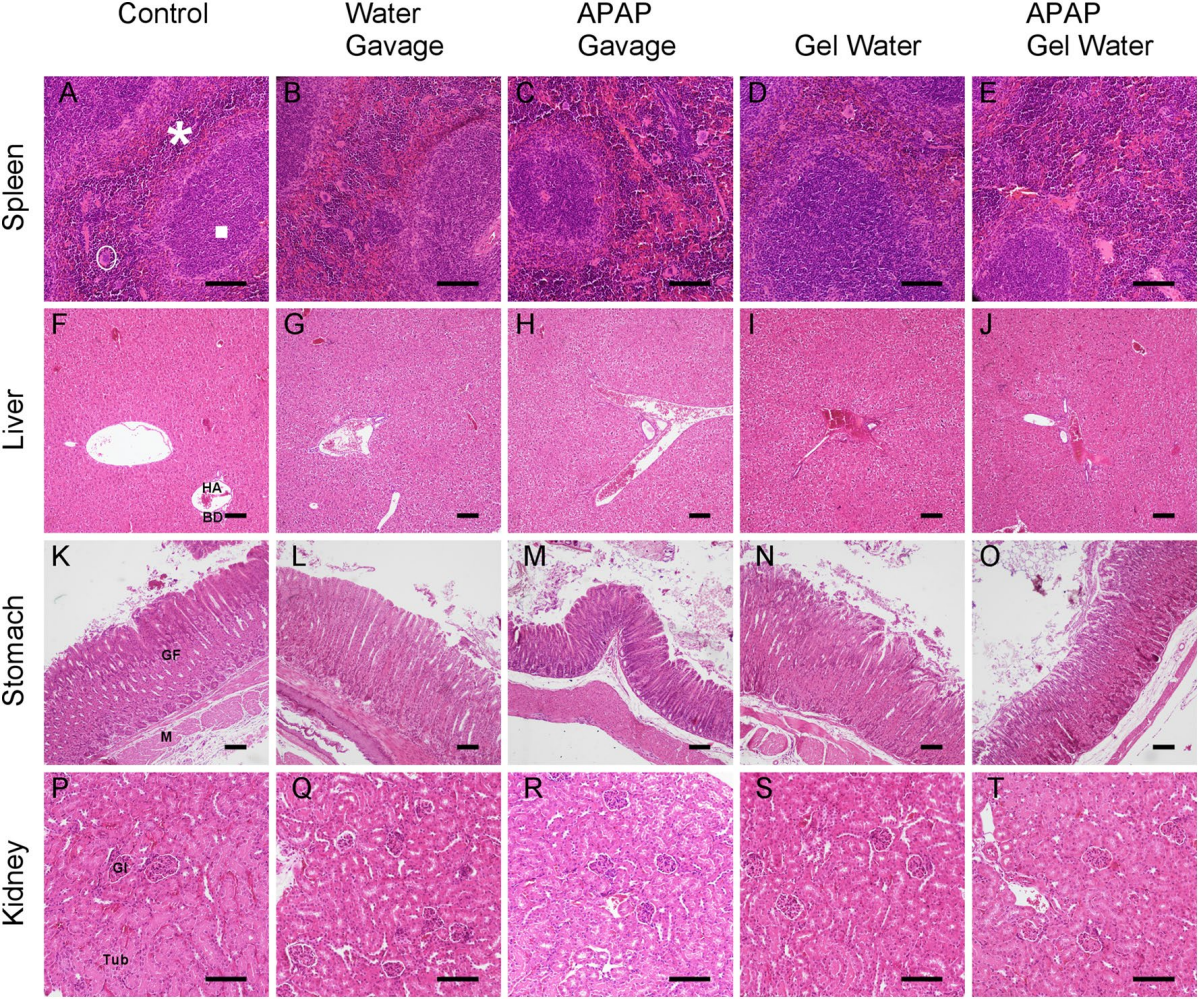
Histological analyses of target tissues (spleen, liver, kidney and stomach) following 5 consecutive days of APAP treatment. Observation after Haemalun-Eosin staining, scale bar: 100 µm. Images (**A**–**E**) section of spleen (white circle: macrophage; square: white pulp; asterisk: red pulp); (**F**–**J**) section of liver (*HA* hepatic artery, *BD* Bile Duct); (**K**–**O**) stomach section (*GF* Fundic Gland, *M* Muscle); (**P**–**T**) section of right kidney (*Gl* tubular Glomerulus, *Tub* collecting tube). (Representative of 4 animals).

To further observed the effect of treatment, histological observations of target tissues (spleen, liver, kidneys, stomach) following treatment were performed. Figure 2 represents a sample of the tissues taken after the APAP treatment on the different groups. Figure 2A,E showed a sample of the spleen of each group. After treatment for 5 consecutive days and regardless of the groups, no change in tissue architecture nor cell infiltration could be observed. Figure 2F–J show the liver for each group. For all groups, no difference between hepatocytes was observed. Indeed, their clarity and the general organization of the tissue is different in the case of hepatotoxicity^31^. No difference in connective tissue containing centrilobular veins was noticeable, just as treatment with APAP did not impact the hepatic lobes surrounding them and the hepatic arteries. No change caused by APAP in the appearance of the stomach layers was observed (Fig. 2K–O). Figure 2P–T represent the sections of the right kidney of the mice in each group. No difference in structure and organization was observed on the two main areas of the kidney: the medulla (central area) and the cortical (peripheral area). For each animal, both kidneys were observed and were not different in their histology.

To further assess the toxicity of APAP, mainly on the liver and its impact on the immune response, the serum IL-6 level was measured on the first and last day of treatment. IL-6 is a good marker of liver toxicity since it is involved in liver regeneration after injury^32^. Thus, IL-6 could not be detected (below the detection threshold of 8 pg/mL) before treatment. No increase was observed after treatment regardless of the group (data not shown).

**Fig. 3:**
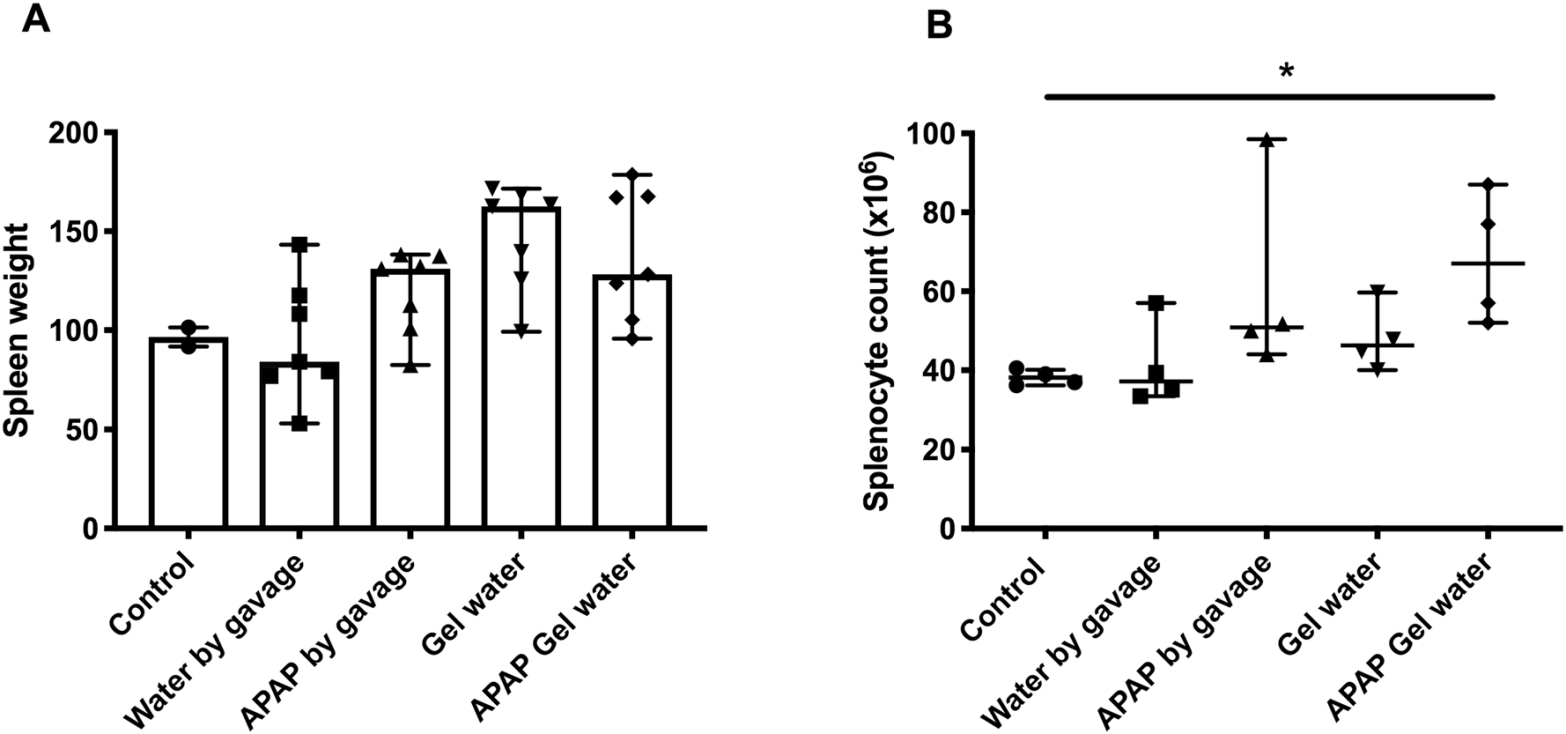
Spleen inflammation after APAP treatment. (**A**) Spleen weight (n = 7, except control n = 2) and (**B**) number of splenocytes (n = 4) after 5 days of consecutive treatment with APAP. Data were analyzed by Kruskal–Wallis test followed by Dunn’s multiple comparisons test and expressed as median ± interquartile range, * indicates P < 0.05.

Spleens were weighted and splenocyte number counted (Fig. 3A,B). No significant difference in spleen weight and splenocytes numbers could be observed between the control groups without treatment and the groups treated with APAP by gavage or self-medication. However, higher splenocyte counts were observed in the groups treated with APAP Gel Water in comparison to the control. The mean percentages of CD4^+^, CD8^+^, CD19^+^ and F4/80^+^ (macrophages) splenocytes were measured. No significant difference could be observed between the groups when compared to the control group (data not shown).

At this dose, APAP treatment did not induce significant changes in zootechnical characteristics, mice behavior and tissue’s structure.

### Effect of APAP treatment on the pathophysiology of toxoplasmosis

#### Zootechnical, clinical and behavioral monitoring of mice

**Fig. 4:**
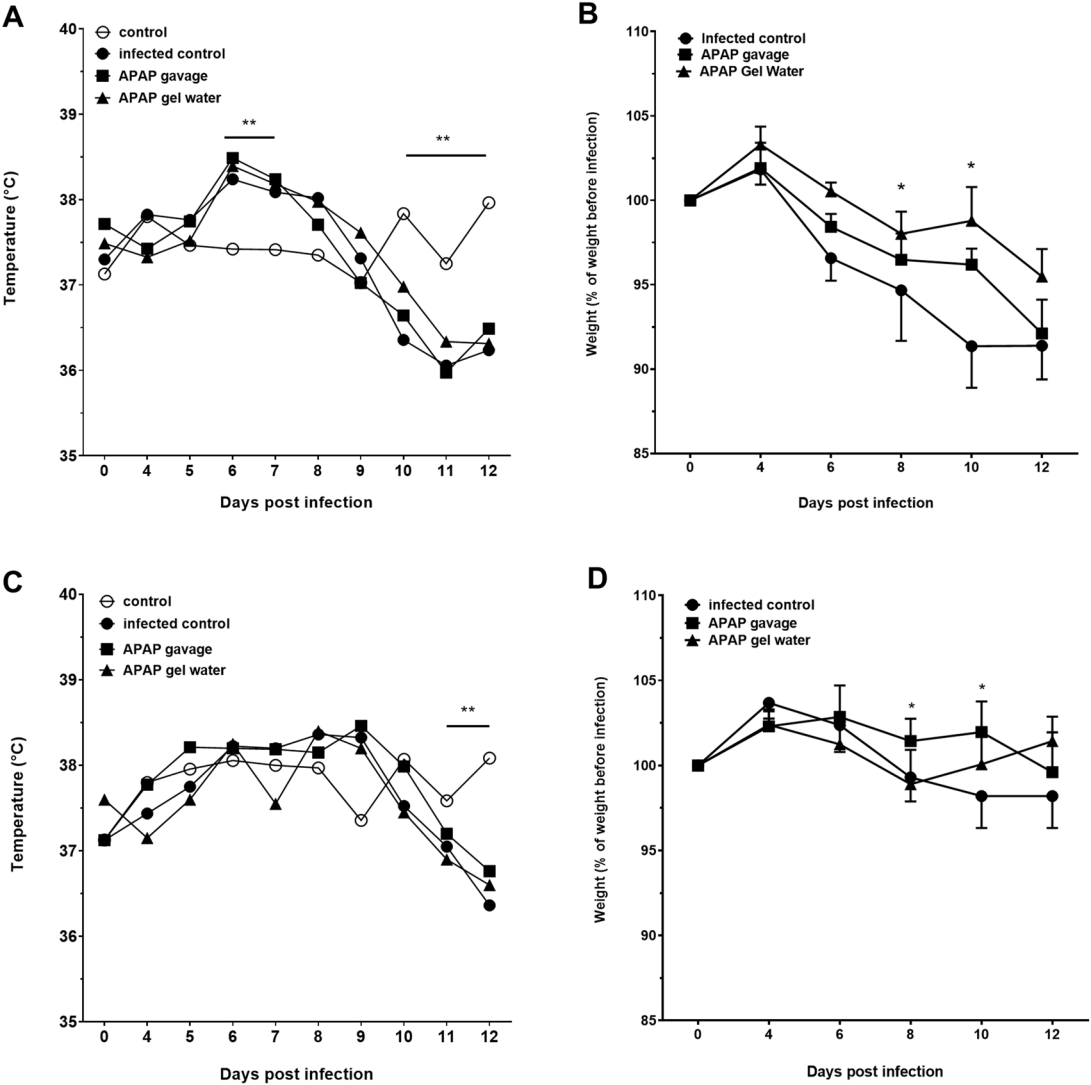
Zootechnical parameters during infection. Monitoring of temperature (**A** and **C**) and weight (**B** and **D**) of mice during the acute phase following infection with 120 cysts (**A** and **B**) or 15 cysts (**C** and **D**), with or without APAP treatment from D7 to D11 (data expressed as mean ± SEM, * indicates P < 0.05, ** P < 0.01, Two-way ANOVA followed by Tukey’s multiple comparisons test, n = 8).

Figure 4 showed the temperature (4A and 4C) and weight (4B, 4D) monitoring of the non-infected and infected mice. The APAP treatment was applied at the beginning of hyperthermic peak of the infected groups. The three groups infected with 120 cysts showed a significant and short hyperthermia compared to the non-infected group at D6 and D7 (Fig. 4A). No significant hyperthermia was observed for the group infected with 15 cysts.

Afterwards, hypothermia was observed until the end of the acute phase (D12) for the 3 infected groups compared to the control non-infected group, independently of APAP treatment. Hypothermia was significant from D10 for the 3 infected groups with 120 cysts and D11 with 15 cysts compared to the uninfected control.

Figure 4B,D showed the weight loss of infected mice compared with their initial weight before infection. The uninfected control group was also monitored during this phase and showed stable weight from D0 to D7 post-infection, followed by a continuous and progressive increase until the end of the study (data not shown).

The three infected groups were compared to see if the expected weight loss was reduced or slowed compared to the untreated infected group. For both infectious doses, weight loss was similar between the three groups and showed no significant difference.

However, looking at each time point the comparison between the 3 groups between D8 and D10, the two treated groups showed a significant difference compared to the infected group, as if the APAP-treated mice had slowed down their weight loss, more so in the higher infectious dose (8–10% weight loss compared with their initial weight). This is in line with what has been described for the acute *T. gondii* infection model in mice. As soon as APAP treatment was stopped (D11), no significant differences were observed between all infected groups.

**Fig. 5:**
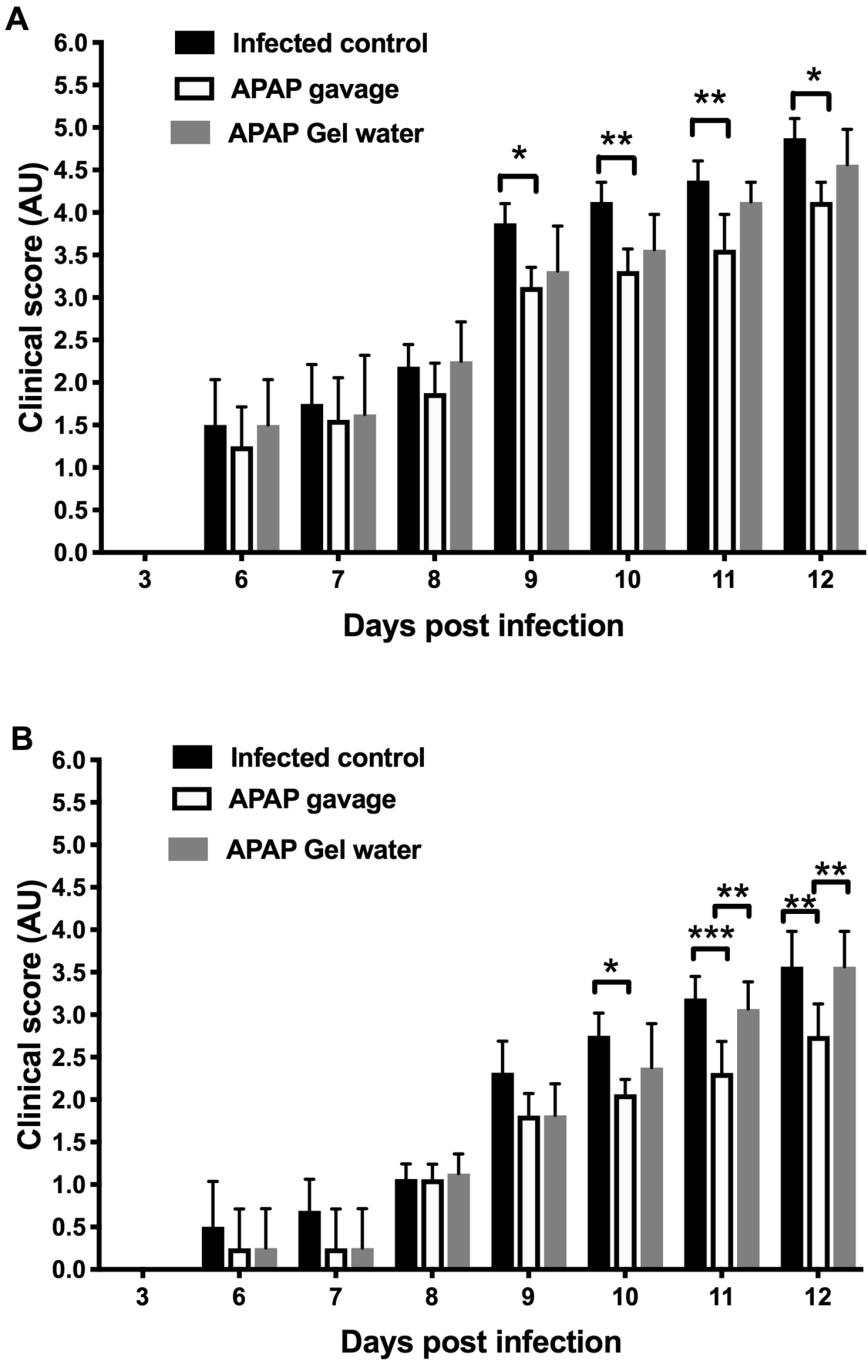
Clinical follow-up. Clinical score during the acute phase following infection with 120 cysts (**A**) or 15 cysts (**B**) with or without APAP treatment from D7 to D11 (data expressed as mean ± SEM, * P < 0.05, ** P < 0.01, *** P < 0.001, Two-way ANOVA followed by Tukey’s multiple comparisons test, n = 8).

To better assess animal pain and a possible effect of APAP during the acute phase of toxoplasmosis, a clinical score was set up with daily observation of the mice. Clinical signs (Table 1) appeared from D6 post-infection and were more important at D12. Between D6 and D10, a change in the overall behavior of the mice with the highest cysts dose was observed (prostration, reduced movement), which was less obvious for the APAP-treated mice. The clinical score was lower in groups infected with 15 cysts than in groups infected with 120 cysts (prostration, hypothermia, reduced movement). For both doses, a clear significant improvement in the score could be observed in the mice treated with APAP by gavage (Fig. 5), from D9 or D10 until D12, one day after the end of treatment. APAP therefore seems to have had a noticeable and significant effect on the well-being of mice, in particular a softening of the face (less tension, normal position of the whiskers), a conservation of social contact between individuals and an increase in mobility.

**Fig. 6:**
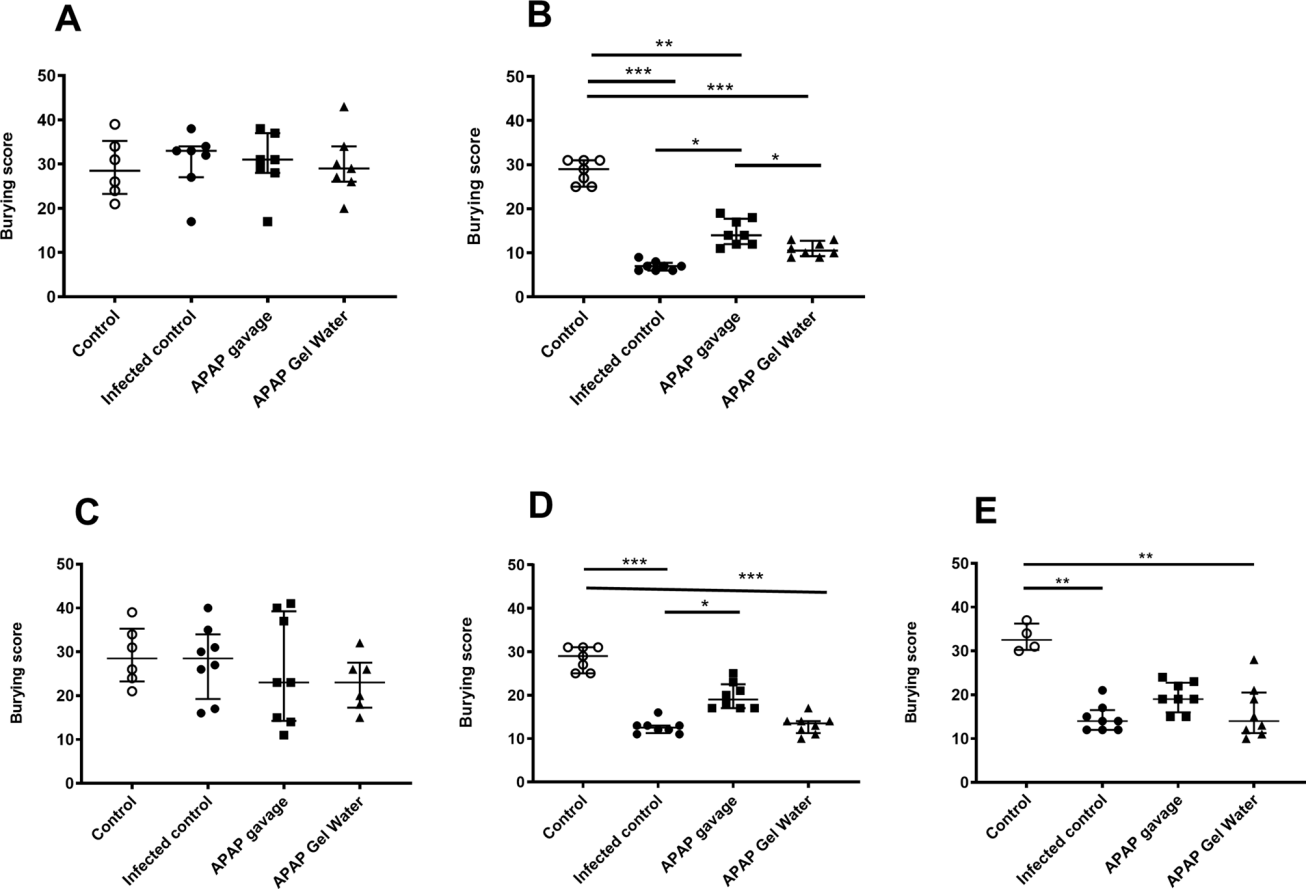
Analysis of behavior during infection. Marble Burying test on D0, D10 after infection with 120 cysts (**A**, **B**) and on D0, D10, D38 after infection with 15 cysts (**C**, **D**, **E**) Data were analyzed by Kruskal–Wallis test followed by Dunn’s multiple comparisons test and expressed as median ± interquartile range, * indicates P < 0.05, ** P < 0.01, *** P < 0.001, n = 8, except control at day 38 n = 4).

Marble burying tests assess mice anxiety score. Animal distress, induced by the animal’s general ill-being, is inversely proportional to the test score. Non-infected controls had a mean score around 30 throughout the experiment (from D0 to D38). Before infection (Fig. 6A,C) and up to D6, no significant difference between groups could be measured, regardless of the infectious dose. For mice infected with 15 cysts, little change in behavior was observed regardless of the group. At D10, the score of the Marble burying test was significantly reduced for all infected groups versus both control groups, with lower scores for mice infected with 120 cysts (mean score of 7, Fig. 6B) than mice infected with 15 cysts (mean score of 10, Fig. 6D). However, an improvement in test scores was observed in infected groups treated with APAP by gavage (Fig. 6B,D), with score around 12 and 20, respectively. The score of chronic dose infected mice remained low compared to the non-infected control group at D21 (Control = 38 ± 4, versus 9 ± 7 for the other 3 groups). A progressive increase in score was observed at D38 (Fig. 6E) to a greater extent in the APAP-treated groups (Control = 33 ± 3, versus infected control 15 ± 3 and infected + APAP 20 ± 3 and infected + APAP + Gel Water 16 ± 6, Fig. 6E).

Altogether these results showed that APAP treatment had an effect on the acute phase of toxoplasmosis for both infectious doses.

To further assess the effect of APAP treatment on infected organs, histological analyses were performed on spleens, lungs and brains.

**Fig. 7:**
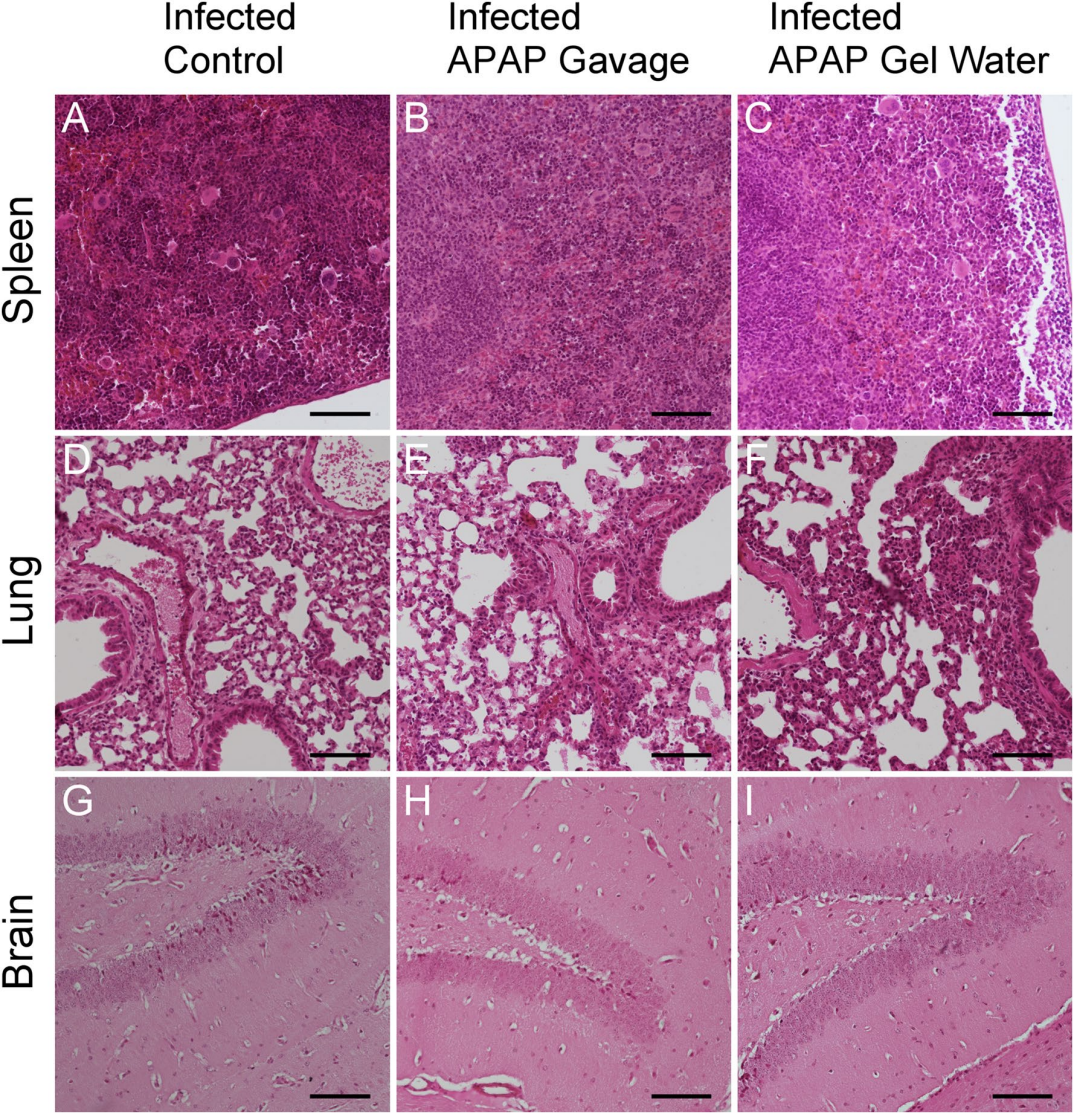
Histological analyses of target tissues during acute phase. Haemalun-Eosin staining of organs from mice of different groups infected with a lethal dose (120 cysts) for 12 days and treated with APAP from D7 to D11, scale bar: 100 µm. Images (**A**–**C**) spleen section; (**D**–**F**) lung section; (**G**–**I**) brain section. Pictures representative of several sections from 3 different animals.

After infection with 120 cysts, significant changes in splenic tissue architecture can be observed, accompanied by massive infiltration of immune cells, such as macrophages, predominantly for the infected group alone. The white pulp is completely unorganized (Fig. 7A,B,C) in the tissue of all infected mice. Likewise, spleen of infected mice appeared to show a higher density of cells, especially at the ends and edges of the splenic capsule. Treatment with APAP does not appear to have altered the inflammatory induction associated with infection with the parasite.

On lung sections of infected mice, an overall thickening of the alveolar epithelium was very clearly observed (Fig. 7D,E,F). The walls of the bronchioles were also thickened by the infection, with no difference observed between the groups that received treatment and the group without treatment. In brain hippocampus sections, astrocytes and neurons could be observed, but no difference in tissue organization (e.g. lesions) between infected groups with or without APAP treatment was induced, (Fig. 7G,H,I). 

**Fig. 8:**
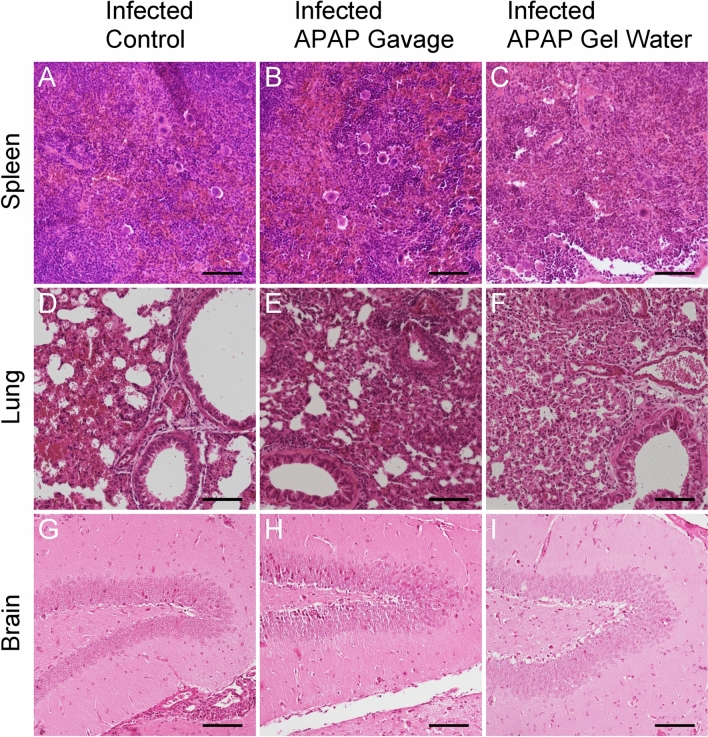
Histological analyses of target tissues during chronic phase. Haemalun-Eosin staining of organs from mice of different groups infected with a chronic dose (15 cysts) for 12 days and treated with APAP from D7 to D11, Scale: 100 µm. Images (**A**–**C**) spleen section; (**D**–**F**) lung section; (**G**–**I**) brain section. Pictures representative of several sections from 3 different animals.

In organ sections from mice infected with 15 cysts, only infiltration of immune cells into the spleen and thickening of the pulmonary epithelium could be observed in all infected groups, treated with APAP or not (Fig. 8). No significant effect of the APAP treatment on the tissue’s lesions had been observed.

#### Cellular and humoral immune responses after infection and APAP treatment

Main cytokines involved in the resolution of infection (IL-12 and IFN γ) were quantified in splenocyte cultures after activation with specific antigens of *T. gondii*. All samples from control non-infected group were under the detection threshold for the cytokines tested.

**Fig. 9:**
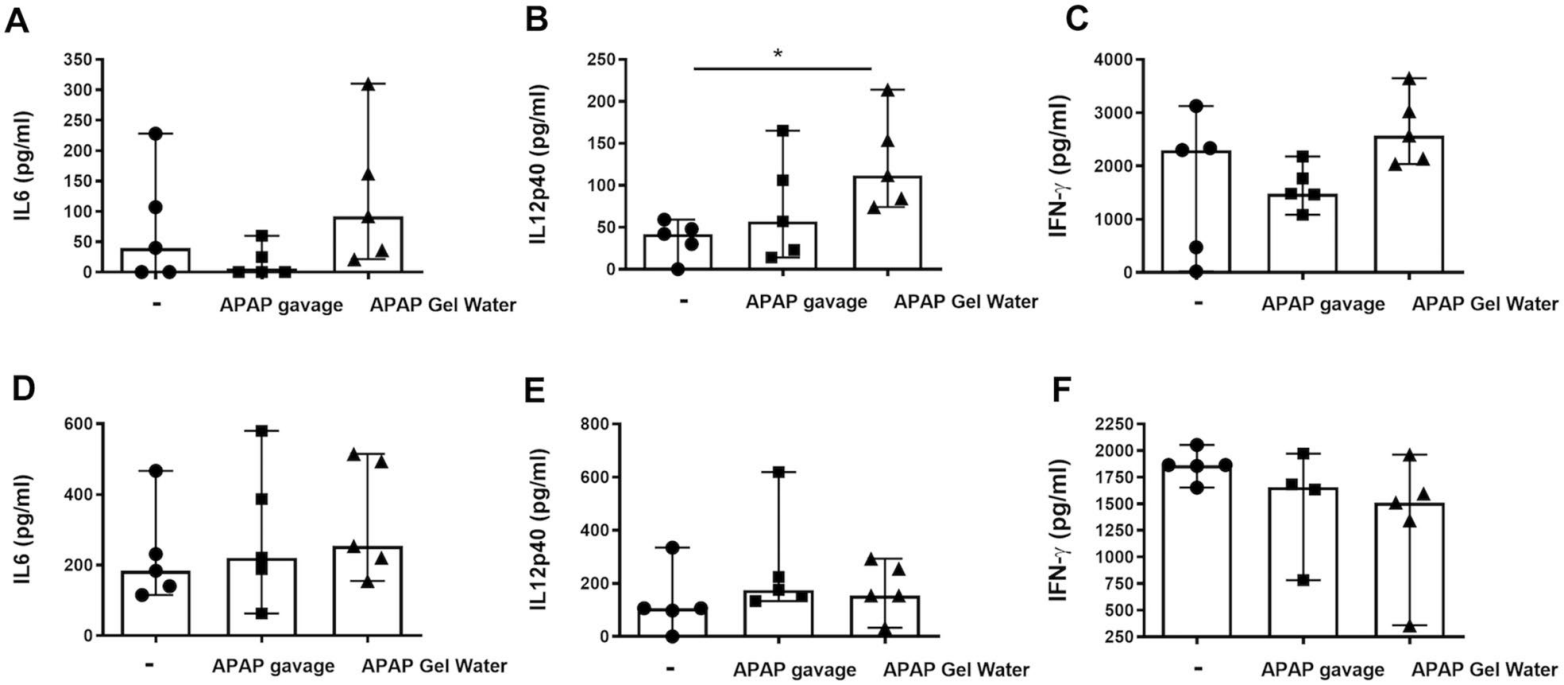
Splenocyte cytokine response. Production of cytokines in supernatants of splenocytes activated in vitro by specific antigens of *T. gondii* from mice after 12 days of infection with 120 cysts (**A**, **B**, **C**) or 42 days of infection with 15 cysts and 5 days of APAP-treatment or without treatment. **A**, **B**, **C** infected with 120 cysts and **D**, **E**, **F** infected with 15 cysts. (**A**) and (**D**) IL-6; (**B**) and (**E**) IL-12; and (**C**) and (**F**) IFN-γ. Data were analyzed by Kruskal–Wallis test followed by Dunn’s multiple comparisons test and are expressed as median ± interquartile range, * P < 0.05, n = 5.

No significant difference in IFN-γ and IL-6 concentrations in the groups treated with APAP compared to the infected group have been shown, regardless the number of cysts (Fig. 9A,C,D,F). For lethal dose groups, IL-12 concentrations were higher in the group treated with APAP in Gel Water compared to the infected group without treatment but not found in the group treated by gavage (Fig. 9B). There was no significant difference in IL-12 concentrations between groups infected with 15 cysts (Fig. 9E).

**Fig. 10:**
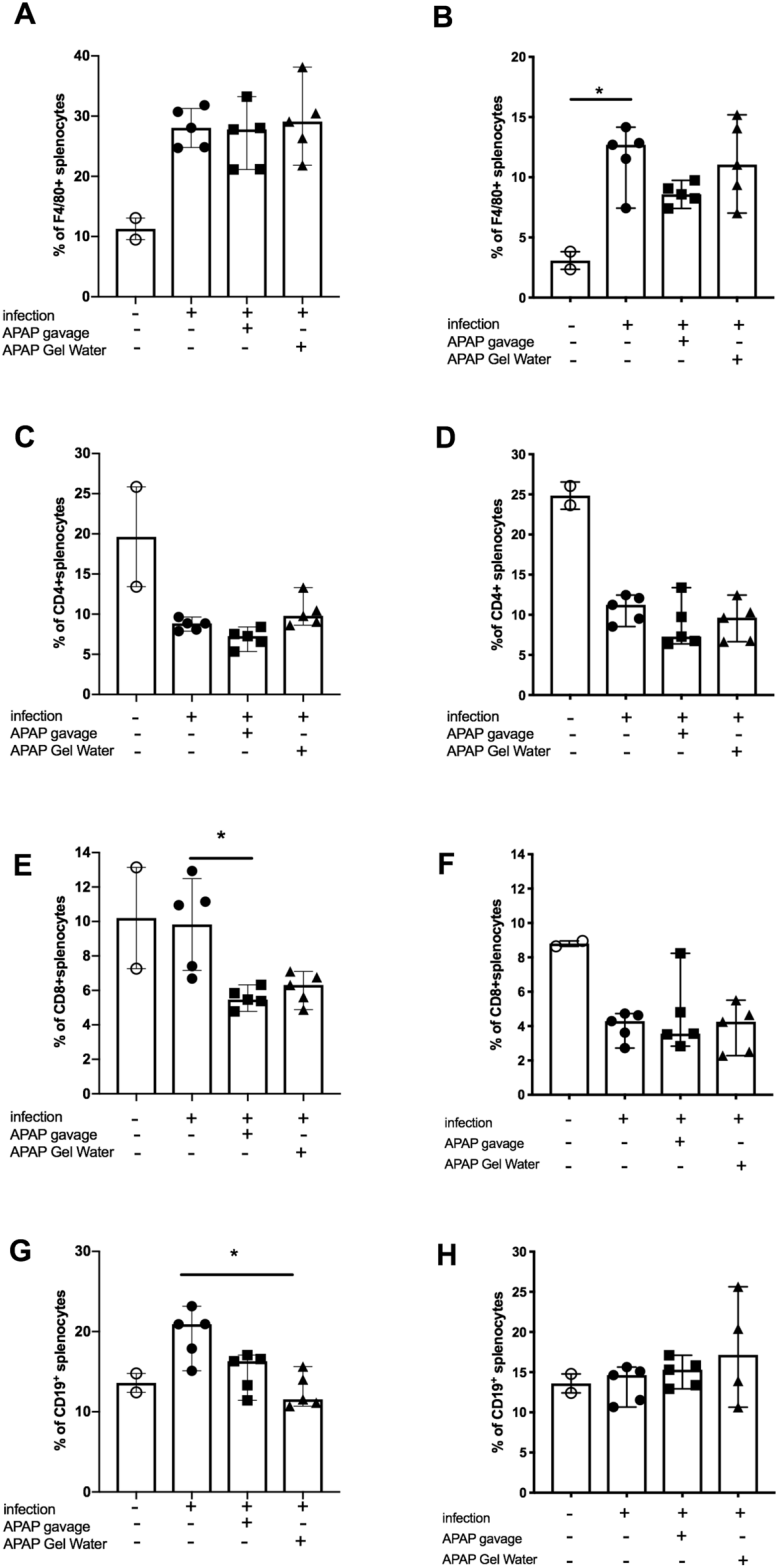
Post-infection distribution of immune splenic populations. Percentage of splenocyte populations after 12 days of infection with 120 cysts (**A**, **C**, **E** and **G**) or 42 days of infection with 15 cysts (**B**, **D**, **F**, **H**) and 5 days of APAP-treatment. A and B: F4/80^+^ (macrophages), **C** and **D**: CD4^+^ T cells; **E** and **F**: CD8^+^ T cells; G and H: CD19^+^ B cells. Data were analyzed by Kruskal–Wallis test followed by Dunn’s multiple comparisons test and are expressed as median ± interquartile range, * P < 0.05, ** P < 0.01, n = 5.

The mean percentages of CD4 + (T lymphocytes), CD8 + (T lymphocytes), CD19 + (B lymphocytes) and F4/80 + (macrophages) populations within the splenocytes were analyzed. Infection had few impacts on populations except for F4/80 + cells. For both infectious doses, percentage of F4/80 + cells increased and this increase is significant for the group infected with 15 cysts (Fig. 10B). After treatment the percentage of F4/80 + , and CD4 + cells did not change (Fig. 10A,C), whereas the percentage of CD8 + cells (Fig. 10E) and CD19 + (Fig. 10G) decreased significantly compared to the infected control group for the treatment by gavage and by Gel water, respectively, for the group infected with 120 cysts. In the mice infected with 15 cysts, (Fig. 10B,D,F,H) the treatment either by gavage or Gel Water had no effect.

In summary, the treatment had a minor impact on population distribution after infection and only on the acute phase of the infection with the high infectious dose.

**Fig. 11:**
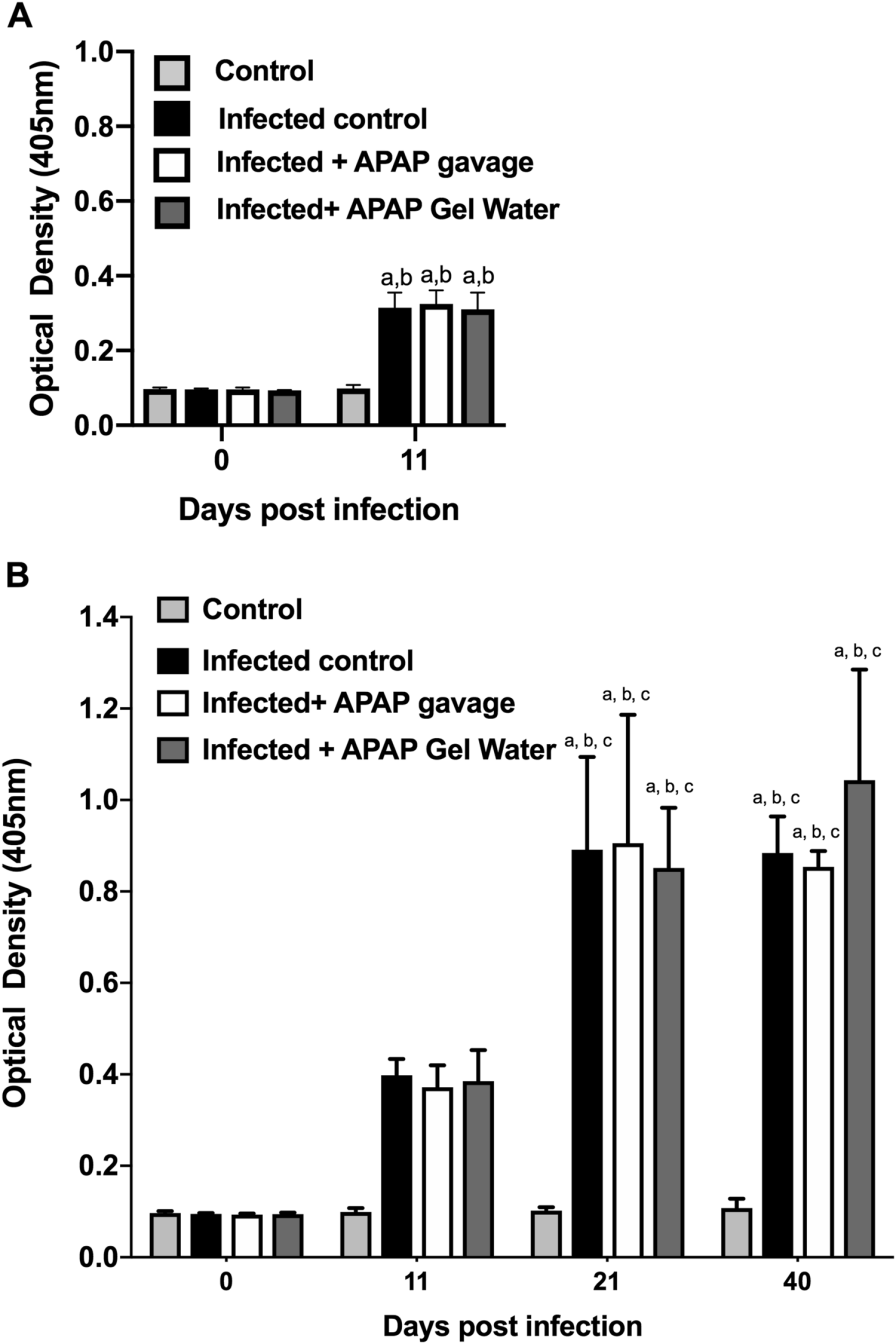
Humoral response after infection. Humoral response in mice infected with 120 cysts (**A**) or 15 cysts (**B**) and APAP-treated (data are expressed as mean ± SD, n = 4). Data were analyzed by two-way ANOVA followed by Tukey’s multiple comparisons test. a **** P < 0.0001 in comparison to day 0, b **** P < 0.0001 in comparison to control group of the same day, c **** P < 0.0001 in comparison to the same group of day 11.

Specific IgG were detected in the sera collected at different times during the experiment to observe seroconversion kinetics following infection (Fig. 11A,B). A significant increase in optical density resulting from infection at D11 for the high dose-infection and at day 21 for the low dose was observed. No difference was observed between the groups treated with APAP and the untreated group, regardless of the infectious dose administered, showing that APAP had no impact on the humoral immune response. Antibody titers have also been performed to get quantitative data and no significant difference between groups was observed (data not shown).

**Fig. 12:**
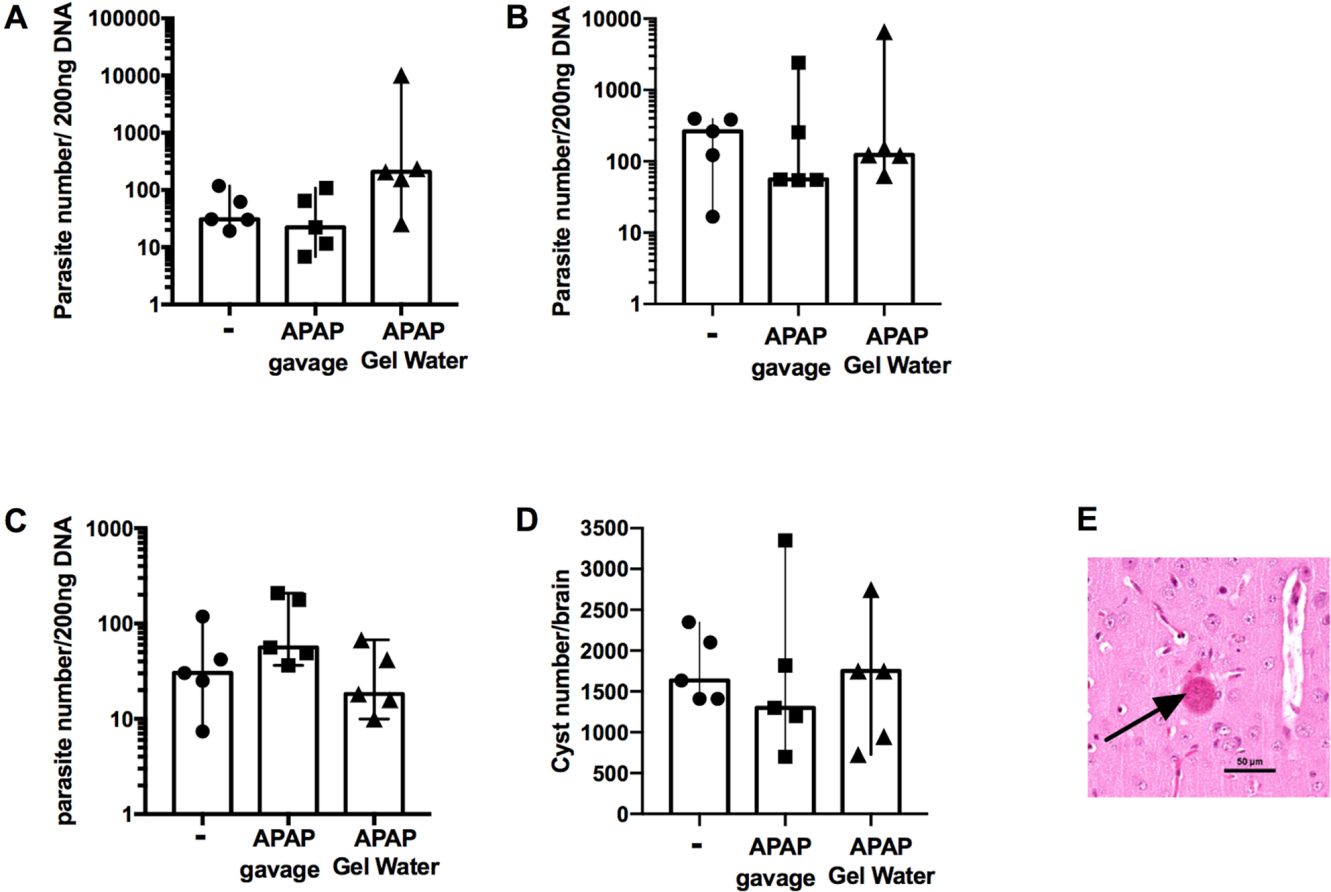
Parasite load during infection with *T. gondii*. A to C *T. gondii* parasite number in 200 ng of total tissue DNA at day 12 after infection with 120 cysts in lung (**A**) and brain (**C**) and at day 42 after infection with 15 cysts in lung (**B**). (**D**) brain cyst counts. Data were analyzed by Kruskal–Wallis test followed by Dunn’s multiple comparisons test and expressed as median ± interquartile rang, n = 5. (**E**) Illustration of cyst in brain.

Parasite load were followed by qPCR in brain and lungs. No significant difference between infected groups and infected groups that received the APAP-treatment (Fig. 12A,B) during the acute phase of infection was observed. In the lungs of the mice in chronic phase, no difference was detected between groups by qPCR (Fig. 12C). Brain cysts of the mice were counted in order to determine the parasite load at the end of the chronic phase (Fig. 12D). The values illustrate high variability between individuals. However, cyst counts did not differ between infected and treated groups.

## Discussion

Pressure around animal experimentation is growing and the most invasive procedures are likely to be controversial and criticized. Animal models are essential in vaccine and therapeutic development against infectious organisms. However, the impact of infections on animals is sometimes severe and leads to problematic clinical signs from an ethical point of view (prostration, long-term hyperthermia, dehydration, even mortality)^33,34^. The use of pharmacological tools to relieve pain remain underutilized in research rodents especially in infectious model despite the general acceptance of both the ethical imperative and regulatory requirements^8,35^. Reticence to use analgesics in animal models may stem from the fear of introducing bias into the model system being analyzed. In way to optimize the conduct of experiments and increase their acceptability in compliance with regulations in animal experimentation focusing on one of the 3Rs, the refinement^36,37^, we used APAP as a new therapeutic refinement tool to relieve the mice in an infectious model of toxoplasmosis.

In the present study, analyzes of the toxicity of APAP showed that its administration had no impact on the weight, temperature and behavior of the mice during the 5-day treatment period. Two routes of oral administration were used, by gavage and by consumption of Gel Water. APAP was administered orally, as its bioavailability and pharmacokinetics are documented by this route of administration^14^ and simpler for use in experimental conditions. This mode of administration has been widely validated by studies on the efficacy of APAP. Administration^38^ by gel consumption is less invasive but the dose of APAP cannot be controlled. No dehydration of the mice was observed when APAP was given in Gel Water suggesting and confirms our previous data^38^. Serum biochemical serum analyzes (ALP, AST, ALAT, urea, glucose) showed that no toxicity was observable. In the case of APAP toxicity, transaminase levels as well as kidneys biomarkers, could increase due to hepatocytes lysis^39,40^, which was not the case here. These results confirm data published on different rodent models, showing that this dose of 30 mg/kg/day is not toxic during 5 consecutive days of treatment^23,40^. The weight of the spleens and the number of splenocytes in non-infected mice were significantly different in the groups having been treated via Gel Water, only for the one with APAP. However, administration of APAP by gavage had no effect on splenocytes number or spleen weight. Gel Water is composed of 99 % water. However, it can be assumed that other components of the Gel such as hydrocolloids (texturizing agent) or fibers (< 1.8%) could have an immunostimulatory effect^41^. No toxicity was observed after histological analyzes on target organs (spleen, liver, kidneys, stomach). This lack of toxicity for this APAP dose is consistent with current published data^23,24^. IL-6 is known to have a dual role in fever regulation and hepatotoxicity after APAP overdose^32^. Serum concentrations of IL-6 were below the limit of detection in all groups. The lack of IL-6, for this dose, confirmed that there is no hepatotoxicity since IL-6 is known to have a role in the regeneration of hepatocytes in case of deregulation and abnormal functioning of the liver^42^. Altogether these data suggest that the dose of 30mg/kg/day of APAP is not toxic as already described in the literature.

The mouse model of toxoplasmosis is well described especially in vaccine trials experiments^43^. Both dissemination and immune responses against the parasite are well known in many mouse strains^44^. Oral infection best represents natural infection and the acute and chronic phases are also well described in the literature. This makes it a good model for infectious disease. The limitation of this model is that infection induces limited symptoms and changes, and only during the acute phase corresponding to parasite dissemination throughout the body.

After *T. gondii* infection, the clinical signs already described for acute toxoplasmosis were recorded (prostration, thermal peaks, face tightness, ears in the back position)^45^.

The first clinical signs observed from the fifth day post-infection were the onset of weight loss. Treatment by both routes reduced weight loss of mice infected with the lethal dose of cysts, probably due to a general improvement in the well-being. This was not so obvious for the mice infected with the lowest dose as weight loss was lower and delayed.

Other symptoms appeared such as prostration, disheveled hair, tightness of face and eye shape, and lack of activity. Self-medication with APAP in Gel Water did not appear to have any noticeable effect on the behavior of the mice, which may be explained by the low daily consumption of gel by the infected mice, especially at the lethal dose of cysts. However, mice infected with 15 cysts were able to ingest enough Gel Water with APAP to get an improvement in their well-being.

A weak hyperthermia peak was observed after infection with the highest dose of cysts and hypothermia was observed at both doses. Although this has never been described in the literature for this infectious model of toxoplasmosis, hypothermia is the most commonly reported predictor of mice imminent death in several infectious model^46^. APAP treatment had low impact on temperature in our experimental conditions.

The dose of APAP could be increased to 100 mg/kg/day as described in various studies^40,47^, but this dose has been shown to induce hepatic toxicity in rodents and pigs after 5 consecutive days^23^.

The detection of mild to moderate pain is difficult to record in mice. Burrowing performance have proved valuable tools to assess brain damage or malfunction^48^. This behavior is reduced by pain and stress, suggesting its use as a behavioral parameter to assess general well-being in mice^49^. As behavior can be observed easily in a non-invasive manner it has been suggested as a relevant approach to assess both pain severity and the efficiency of pain management drugs. We used the Marble Burying Test to evaluate pain and pain relief after treatment.

This test showed a marked decrease in the activity of stressed or prostrated mice due to general unease during acute toxoplasmosis at both infectious doses. This is entirely consistent with the clinical signs observed. Other types of behavior tests could be used, such as the cross maze which could be used to study the exploration of a new environment like the Marble Burying test does, although the latter makes it easier to quantify anxiety^24^. On the other hand, in our study, a clear and significant improvement in the clinical signs and the general behavior of the mice was observed on the 3rd day of treatment with APAP for both infectious doses. The hypothesis is that these two parameters may be related. Indeed, the clinical score and the behavioral tests may be linked because a mouse with reduced mobility due to symptoms will naturally bury fewer marbles than a healthy mouse. A higher score of the Marble Burying test seems to illustrate an increase in the locomotion of the mice in relation to the general well-being effect induced by APAP, which also allowed the mice to be able to continue eating and hydrating (decrease of weight loss in mice treated by gavage) at both infectious doses. Similar results were observed in the literature, on a non-infectious mouse model^50^. This is a positive point for the use of APAP by gavage mainly to relieve the animal’s discomfort. These first results are completely innovative in murine toxoplasmosis with these behavioral approaches in an infectious process and are consistent with the known effect of APAP on the general improvement of the individual^23^. One publication describes the use of buprenorphine to relieve pain and distress after acute *T. gondii* infection^21^. They only observed time to death after infection by injection with a virulent strain and relief of pain distress. They didn’t look at parasite dissemination or the immune response.

Analysis of immune populations (CD4 + , CD8 + and CD19 + lymphocytes, macrophages), showed no significant differences between the infected groups except for CD8 + T cells and CD19 + B cells, which were lower in the APAP gavage and gel groups, respectively, than in the infected control group. Lymphocyte depletion in lymphoid organs due to APAP has already been observed, but at high doses and it was associated with hepatotoxicity^45,51^. Cytokines of interest were measured: IL-12 and IFN-γ as primary responses to *T. gondii* infection, IL-6 for its role in fever regulation and hepatotoxicity. The levels of these cytokines were slightly modified after stimulation of splenocytes with specific antigens. A non-significant decrease in the IL-6 concentration could be observed in the APAP-gavage treated group. Fever is partly regulated by IL-6^22,23^. However, APAP is an antipyretic and even if it has no direct effect on the amounts of IL-6, it could induce a change in its concentration^52^. The concentration of IL12 was also significantly increased in the group treated with APAP in Gel Water in the group infected with the highest dose of cysts. This may be consistent with the observation of a potential immunostimulatory effect due to the component of the gel^41^. 

Specific serum IgGs levels were not modified for both infectious doses by treatment with APAP. This indicates that APAP had a weak impact on the cellular response and no effect on humoral immune response in our experimental conditions.

Parasite load was assessed by qPCR for organs obtained at the end of acute phase (lungs, brains) and at the end of the chronic phase (lungs) and also by counting the brain cysts. No significant difference in parasite load was observed in either tissue regardless of the treatment. Related to brain and chronic phase behavior, no difference in cyst number was observed, suggesting that APAP may improve behavior by reducing symptoms without affecting parasite load in the mice, showing a very limited effect on the physiopathology. Finally, the histological analysis showed no difference in the tissue organization of the target organs due to the treatment, especially in the brain hippocampus region which is involved in the behavior and the exploration of a new environment. Since *T. gondii* is known to induce changes in behavior when installed in the brain, it was interesting to study the relationship between APAP treatment and behavior. Indeed, it was important that improvement in behavior during both phases of the infection was due to APAP reducing the severity of symptoms rather than by modifying the parasite load. It is also important to note that APAP’s mode of action could be linked with serotonin levels^53^, one of the main mediators of anxiety in the central nervous system^54^.

## Conclusion

APAP administered by gavage seems to be a good pharmacological tool to relieve an animal infected with *T. gondii* and thus refine the infectious process by improving well-being, as shown here in the model species. Our project therefore involved highlighting the combination of individual or collective markers specific to toxoplasmosis in mice (zootechnical, behavioral, immune response, parasite dissemination). The application of APAP during the acute phase did not modify all these parameters related to the pathophysiology of murine toxoplasmosis. However, this treatment significantly improved the general condition of the mice, and thus contributed to the animal’s well-being during experimentation. These initial results confirm our hypothesis and validate our proof of concept that APAP, at this reference dose and in our infectious model, is a new pharmacological tool for experimentation in infectiology to improve animal welfare. From a scientific and academic point of view, these research studies will also lead to a better understanding of the mode of action of APAP in veterinary medicine and in host–pathogen interactions, without altering pathophysiology and improving animal welfare. This work will perhaps make fall a dogma on the use of APAP in veterinary health from animal experiments to breeding.

## Data Availability

The datasets used and/or analysed during the current study available from the corresponding author on reasonable request. All data generated or analysed during this study are included in this published article.
